# Daily Associations of Neighborhood Quality and Cognitive Function

**DOI:** 10.1093/geroni/igaf122.1176

**Published:** 2025-12-31

**Authors:** Alexa Allan, Roland Thorpe, Jr., Lesley Ross, Christopher Engeland, Orfeu Buxton, Alyssa Gamaldo

**Affiliations:** The Pennsylvania State University, University Park, Pennsylvania, United States; Johns Hopkins University, Baltimore, Maryland, United States; Clemson University, Clemson, South Carolina, United States; The Pennsylvania State University, University Park, Pennsylvania, United States; The Pennsylvania State University, University Park, Pennsylvania, United States; Clemson University, Clemson, South Carolina, United States

## Abstract

Research has indicated that measures of positive and negative neighborhood quality relate to cognitive function in opposing ways. However, limited studies have examined the role that daily reports of neighborhood quality may play in daily cognitive functioning. Thus, this study examined the daily associations between neighborhood quality and cognitive function in a sample of Black and White adults from the HANDLSleep study in Baltimore, MD. Measures of neighborhood quality (e.g., litter, buildings being repaired) and cognitive function (Symbol Search [processing speed] and Dot Memory [working memory]) were collected using daily mobile phone surveys over a 7-day period. Multilevel linear models tested the within- and between-person associations among reported positive and negative neighborhood characteristics and cognitive function, adjusted for age, sex, race, reading literacy, poverty status, depressive symptomology, and medical condition history. The analysis included 229 midlife to older adults (Mage = 62.65, SDage = 8.63; 72% female; 66% Black). Of the 18 items, on average, participants reported 1.73 (SD = 2.52) and 1.34 (SD = 2.93) positive and negative neighborhood characteristics, respectively. ICC results revealed that 59.7% of the variance in reports of positive neighborhood characteristics and 39.9% of variance in reports of negative neighborhood characteristics could be attributed to within-person differences. No significant associations were observed between positive and negative neighborhood characteristics and cognitive function. While significant associations were not observed, the findings underscore the importance of considering methodology when capturing between- and within-person variability in neighborhood characteristics within aging adults.

